# The origins of binding specificity of a lanthanide ion binding peptide

**DOI:** 10.1038/s41598-020-76527-y

**Published:** 2020-11-10

**Authors:** Takaaki Hatanaka, Nobuaki Kikkawa, Akimasa Matsugami, Yoichi Hosokawa, Fumiaki Hayashi, Nobuhiro Ishida

**Affiliations:** 1 Toyota Central R&D Labs., Inc., 41-1, Nagakute, Aichi 480-1192 Japan; 2 Advanced NMR Application and Platform Team, NMR Research and Collaboration Group, NMR Science and Development Division, RIKEN SPring-8 Center, 1-7-22 Suehiro-cho, Tsurumi-ku, Yokohama, Kanagawa 230-0045 Japan

**Keywords:** Biophysics, Computational biology and bioinformatics, Structural biology, Coordination chemistry, Inorganic chemistry

## Abstract

Lanthanide ions (Ln^3+^) show similar physicochemical properties in aqueous solutions, wherein they exist as + 3 cations and exhibit ionic radii differences of less than 0.26 Å. A flexible linear peptide lanthanide binding tag (LBT), which recognizes a series of 15 Ln^3+^, shows an interesting characteristic in binding specificity, i.e., binding affinity biphasically changes with an increase in the atomic number, and shows a greater than 60-fold affinity difference between the highest and lowest values. Herein, by combining experimental and computational investigations, we gain deep insight into the reaction mechanism underlying the specificity of LBT3, an LBT mutant, toward Ln^3+^. Our results clearly show that LBT3-Ln^3+^ binding can be divided into three, and the large affinity difference is based on the ability of Ln^3+^ in a complex to be directly coordinated with a water molecule. When the LBT3 recognizes a Ln^3+^ with a larger ionic radius (La^3+^ to  Sm^3+^), a water molecule can interact with Ln^3+^ directly. This extra water molecule infiltrates the complex and induces dissociation of the Asn5 sidechain (one of the coordinates) from Ln^3+^, resulting in a destabilizing complex and low affinity. Conversely, with recognition of smaller Ln^3+^ (Sm^3+^ to Yb^3+^), the LBT3 completely surrounds the ions and constructs a stable high affinity complex. Moreover, when the LBT3 recognizes the smallest Ln^3+^, namely Lu^3+^, although it completely surrounds Lu^3+^, an entropically unfavorable phenomenon specifically occurs, resulting in lower affinity than that of Yb^3+^. Our findings will be useful for the design of molecules that enable the distinction of sub-angstrom size differences.

## Introduction

Lanthanide elements (Ln) consist of 15 elements with similar physicochemical properties; they exist as + 3 cations (Ln^3+^) in solution and exhibit ionic radii differences of less than 0.26 Å^1^. Because of their unique magnetic and optical properties, Ln-element-based compounds are applied in various advanced materials, such as rechargeable batteries, lamp phosphors, and permanent magnets^2,3^. For sustainable usage and advancement of Ln elements, the construction of more efficient recovery techniques and recycling methods is required^4,5^. Therefore, many researchers have attempted to find molecules with recognition ability toward each Ln element^6–8^. For example, some chelating agents [e.g., ethylenediaminetetraacetic acid (EDTA) and nitrilotriacetic acid (NTA)] have been demonstrated to bind with Ln^3+^, and their affinity gradually increases as the ionic radius of Ln^3+^ decreases (Supplemental Fig. S1a,b)^9,10^. This gradual increase in affinity is simply explained by the trend of increasing Ln^3+^ acidity with decreasing ionic radius. However, most of these molecules lack the recognition specificity for clearly identifying individual Ln^3+^ elements.

A linear peptide consisting of 17 amino acids, named lanthanide binding tag (LBT), shows an interesting recognition pattern for Ln^3+^^11^. This peptide originates from a calcium-binding protein, calmodulin, and has been engineered to recognize Ln^3+^ by combinatorial screening^12,13^. Because of the short peptide sequence and high binding affinity, LBT has been incorporated into recombinant proteins and used as an effective tool for structural analysis^14–17^. In particular, the binding affinity of LBTv, one of the LBT variants, along the Ln^3+^ series does not correlate simply to the decrease in the ionic radii (Supplemental Fig. S1c); a biphasic change occurs with Eu^3+^ as the branching point and shows drastically different binding affinities toward different Ln^3+^, exhibiting a > 60-fold lower affinity for La^3+^ than for Tb^3+^, with KD values of 3500 nM and 57 nM, respectively^11^. These binding properties indicate that a different mechanism underlies the recognition specificity of LBT compared to that of other chelating agents such as EDTA and NTA; however, the origin of this recognition specificity has not yet been investigated. The elucidation of the recognition mechanism of LBT toward Ln^3+^ would greatly contribute to the development of specific agents for Ln^3+^ recovery as well as for the accurate artificial design of functional peptides.

Herein, to understand the mechanism of LBT specificity, we evaluated the thermodynamic parameters of binding between LBT3, an LBT derivative exhibiting the highest binding affinity to Ln^3+^^16^, and Ln^3+^. We further elucidated the structures of LBT3-Ln complexes in solution. In addition, the structural fluctuations and the interactions between the complexes and the surrounding water molecules were analyzed using molecular dynamics (MD) simulations. Our results showed that the structures of the LBT3-Ln complexes for different Ln^3+^ are similar. However, the thermodynamic parameters (Δ*H* and Δ*S*) changed intricately across the series of Ln^3+^, which can be divided into three categories, namely, La^3+^ to Sm^3+^, Sm^3+^ to Yb^3+^, and Lu^3+^. In addition, we clarified that the large affinity difference is attributed to a water molecule, which directly coordinates with Ln^3+^ in an LBT3-Ln complex.

## Results

### Thermodynamic parameters of LBT3 binding with Ln^3+^

**Figure 1:**
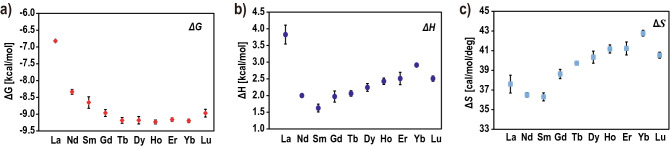
Thermodynamic parameters of the binding between LBT3 and a series of Ln^3+^. (**a**) The change in free energy. (**b**) The change in enthalpy. (**c**) The change in entropy. All experiments were performed at least three times. The error bars indicate the standard error.

Isothermal titration calorimetry (ITC) experiments were performed to evaluate the thermodynamic parameters of the binding of LBT3 with a series of Ln^3+^ (Fig. 1, Supplemental Table S1). All experiments were performed at pH 6.0, as some Ln^3+^ easily form hydroxide species and precipitate above pH 7.0^9^. All the reactions showed endothermic behavior; the change in free energy (Δ*G*) decreased with increasing Ln^3+^ atomic number, from La^3+^ (Δ*G* = -6.9 kcal/mol) to Tb^3+^ (Δ*G* = -9.1 kcal/mol). The Δ*G* then remained almost constant from Tb^3+^ to Yb^3+^, and then increased slightly for Lu^3+^. Due to the differences in measurement conditions, especially pH and temperature, the current affinity values show an approximately tenfold difference compared to those in previous reports using the same LBT3^13,16^. However, the relative affinities for each Ln^3+^ were the same as those of LBTv (Supplemental Fig. S1c)^11^.

The changes in the enthalpy (Δ*H*) and entropy (Δ*S*) of binding with each Ln^3+^ followed a different pattern than that for Δ*G*; both parameters decreased from La^3+^ to Sm^3+^, increased from Sm^3+^ to Yb^3+^, and finally decreased again for Lu^3+^. These results indicate that the difference in affinity is not solely dependent on the acidity of the Ln^3+^, but could be influenced by multiple factors, such as the ion size, hydration number, and acidity of the Ln^3+^. Moreover, we surmised that these differences might be partially responsible for the structural differences among the LBT3-Ln complexes.

### Differences between the ^1^H NMR spectra of the LBT3-La and LBT3-Lu complexes

**Figure 2:**
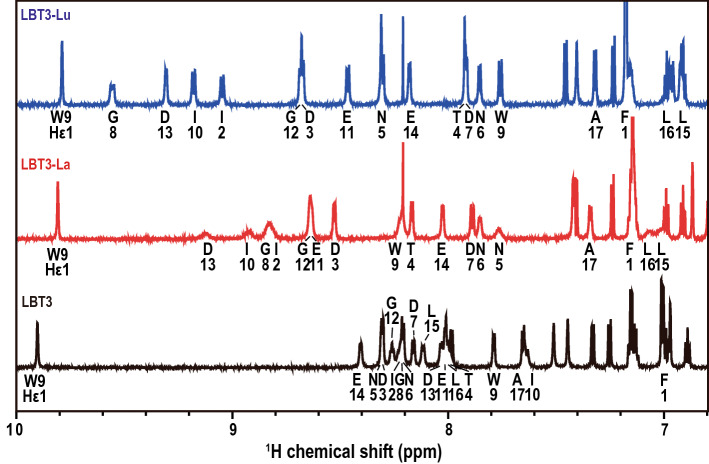
NMR spectra of LBT3-Ln complexes. ^1^H NMR spectra of the amide proton region of LBT3, LBT3-La, and LBT3-Lu obtained using 100 µM LBT3 or 100 µM LBT3 with 200 µM La^3+^ or Lu^3+^. The amino acid assignments are based on standard homonuclear two-dimensional NMR methodology^31^.

To compare the structures of a strongly bound and weakly bound complex, La^3+^ and Lu^3+^ were chosen for nuclear magnetic resonance (NMR) experiments. LBT3 exhibits a greater than 40-fold difference in affinity toward La^3+^ and Lu^3+^ under our conditions (Supplemental Table S1)^11^, which might reflect a difference in the LBT3-Ln structures. Moreover, these two ions are diamagnetic, which enables a straightforward comparison of their NMR spectra. The other Ln^3+^ in the series are paramagnetic, which leads to drastic changes in chemical shifts and peak shapes^18^. As shown in Fig. 2, there are obvious differences between the ^1^H NMR spectra of the LBT3-La and LBT3-Lu complexes; LBT3-Lu displays sharp peaks, while LBT3-La exhibits broadened peaks; large chemical shift differences were observed in the amide protons of N5 (N5-HN) and G8 (G8-HN), of 0.5 ppm and 0.7 ppm, respectively (Supplemental Fig. S2). Upon titration, we observed a clear difference between the exchange rates of the binding of LBT3 with La^3+^ and Lu^3+^; a slow exchange was observed for Lu^3+^, and an intermediate exchange was observed for La^3+^ (Supplemental Fig. S3). Titration of Lu^3+^ into the LBT3 solution resulted in the immediate formation of new peaks related to complexation, while the peak intensity of free LBT3 decreased as the Lu^3+^ concentration increased. The free LBT3 peak disappeared upon the addition of a two-fold Lu^3+^ concentration. Conversely, titration with La^3+^ initially induced a decrease in the intensity of the free LBT3 peaks. An unknown peak was observed at LBT3:La = 1:1, and peaks associated with complex formation resolved at LBT3:La = 1:2. Variable temperature ^1^H NMR measurements also showed that the LBT3-La spectrum sharpened at higher temperatures, indicating an intermediate to fast exchange, while the LBT3-Lu peaks became broad, indicating a slow to intermediate exchange (Supplemental Fig. S4).

### Structures of LBT3-Ln complexes

**Figure 3:**
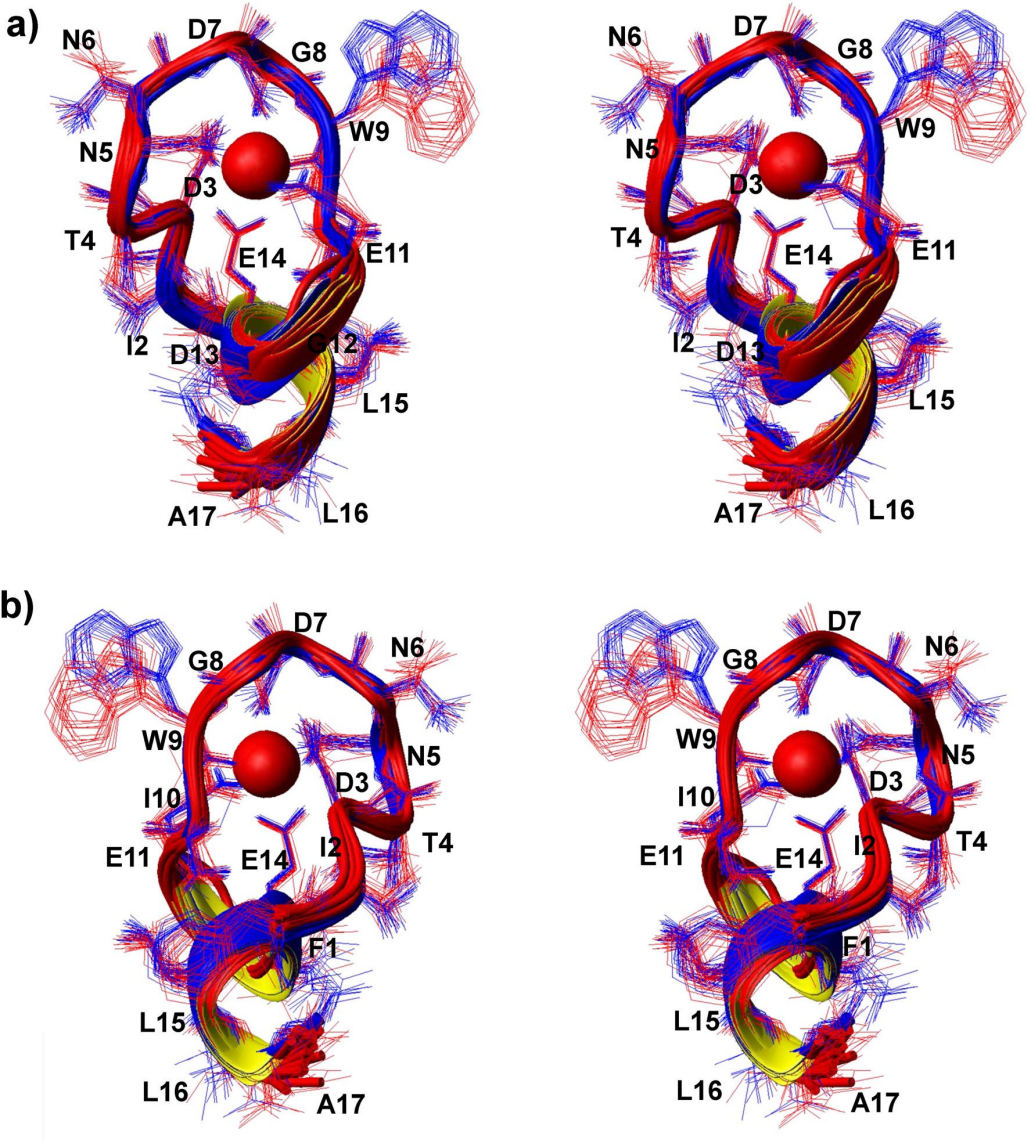
Stereo view of the complex structures of 6LBT3-La and 6LBT3-Lu. (**a**) 6LBT3-La (red) and 6LBT3-Lu (blue) are superimposed. The sphere represents Ln^3+^. (**b**) 180° rotated view of (**a**). 20 structures of each 6LBT3-Ln complex are superimposed. The sidechain hydrogens are omitted for clarity.

To elucidate the structures of LBT3-Ln complexes in aqueous solution, gel-filtration chromatography was first performed to determine the self-assembled state at pH 6.0. As a result, free LBT3 showed a clear single peak at the three concentrations tested, and no differences were observed in the retention volume (Supplemental Fig. S5a). Moreover, the complexes LBT3-La and LBT3-Lu both showed clear single peaks and larger retention volumes, compared to free LBT3 (Supplemental Fig. S5b). These results indicate that the free LBT3 and LBT3-Ln complexes exist as monomers in solution at pH 6.0. Next, to examine the binding site, ^1^H NMR analyses were performed using Sm^3+^, a Ln^3+^ with relatively weak paramagnetism. Large paramagnetic shifts were observed at D3, N5 to E11, and E14 (Supplemental Fig. S6); these results are in good agreement with previous X-ray crystallographic structure or mutation analyses, in which the LBT series peptides were found to surround the Ln^3+^ with residues D3 to E14 and bind to Ln^3+^ via the side chain carboxylates of D3, D7, E11, E14, the side chain carbonyl oxygen of N5 (N5-Oδ1), and the backbone carbonyl oxygen of W9 (W9O). Solution structures were calculated using the program CYANA based on the NOESY spectra of free LBT3, LBT3-La, and LBT3-Lu measured at 10 °C. All structures were calculated as monomers, and LBT3-La and LBT3-Lu were calculated using the condition that Ln^3+^ interacts with the six sites mentioned above. The calculated complex structures were referred to as 6LBT3-La and 6LBT3-Lu. Free LBT3 exhibited a random structure, while 6LBT3-La and 6LBT3-Lu showed highly fixed structures (Fig. 3, Supplemental Fig. S7). A comparison of the dihedral angles and chi1 angles of these two complexes showed strong similarity (Supplemental Fig. S8a–c), and the backbone root mean square distribution (rmsd) was calculated as 0.354 Å. In addition, the obtained structures were fairly similar to the previously reported crystal structure of LBTv-Tb (Supplemental Fig. S8d); the calculated backbone rmsd value is 0.412 Å. The strong similarity with LBTv-Tb firmly supports the reliability of our structural calculations, although unexpectedly, we could not identify any notable differences between the 6LBT3-La and 6LBT3-Lu structures.

### N5-Oδ1 dissociation from Ln^3+^

**Figure 4:**
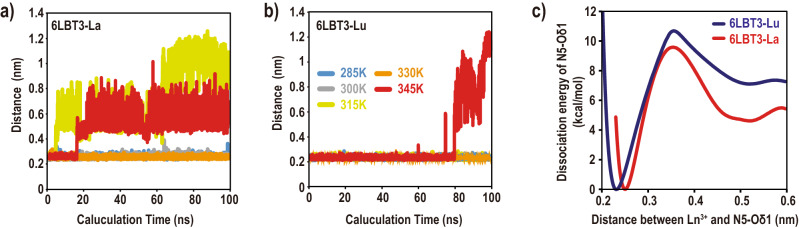
Dissociation of N5-Oδ1 from Ln^3+^ observed in MD simulations. (**a**,**b**) Changes in distances between Ln^3+^ and the coordinated carbonyl oxygen N5-Oδ1 observed during 100 ns of MD simulation for (**a**) 6LBT3-La and (**b**) 6LBT3-Lu. Simulations were conducted at different temperatures (285 K to 345 K), as indicated in (**b**). (**c**) Free energy landscapes of coordination/dissociation reactions between N5-Oδ1 and Ln^3+^. Roughly, when the distance between Ln^3+^ and N5-Oδ1 is < 0.3 nm, N5-Oδ1 is coordinated with Ln^3+^. Conversely, when the distance is > 0.4 nm, N5-Oδ1 is dissociated from Ln^3+^.

To investigate the differences between 6LBT3-Lu and 6LBT3-La, all-atom MD simulations of these complexes were performed at various temperatures, from 285 to 345 K, for 100 ns each without any constraints (see the Experimental Section for details) using GROMACS software^19^. We performed five different types of simulations for each complex, and found that N5-Oδ1 dissociates from Ln^3+^ more easily than the other coordinated residues. This dissociation is considerable in the case of 6LBT3-La. In this system, the distance between N5-Oδ1 and La^3+^ rapidly increased from ~ 0.2 nm to > 0.4 nm for the 315 K and 345 K calculations (Fig. 4a, Supplemental Fig. S9a). On the other hand, in the 6LBT3-Lu system, the distance between N5-Oδ1 and Lu^3+^ remained stable at 0.2 nm, except during the last phase at 345 K, in which the distance increased to > 0.4 nm after 80 ns of calculation (Fig. 4b, Supplemental Fig. S9b). All the other coordinated residues consistently chelated Ln^3+^ throughout the calculations in both complexes (Supplemental Fig. S9c,d). Although the N5-Oδ1 dissociation is only observed in 3 out of 10 conditions examined here (possibly due to the slightly shorter calculation time), these results, at least, indicate that the binding between N5-Oδ1 and Ln^3+^ was considerably weaker than that of the other coordinating residues, especially in the case of La^3+^. This is reasonable because this dipole-ion interaction is weaker than the ion-ion interactions. Although the continuously bound site W9O also coordinated Ln^3+^ via dipole-ion interactions, W9O is tightly structurally restrained compared to the other residues.

The free energy landscape as a function of the distance between N5-Oδ1 and Ln^3+^ was calculated using the accelerated weight histogram (AWH) method for 200 ns each at 300 K^20^. To distinguish the association/dissociation effect of N5-Oδ1 from the effect of a large overall structural change in LBT3, 0.5 kcal/mol/Å^2^ harmonic constraints were applied to the backbones during the free energy calculations. Remarkable differences were observed between the free energy landscapes of the 6LBT3-La and 6LBT3-Lu systems. In the 6LBT3-Lu system, the associated state is 7.1 kcal/mol more stable than the dissociated state, whereas in 6LBT3-La, the associated state was only 4.6 kcal/mol more stable than the dissociated state (Fig. 4c). Although artificial constraints were applied in these simulations, these calculations also indicated that the dissociation of N5-Oδ1 occurs more easily in LBT3-La than in LBT3-Lu.

### Monodentate or bidentate chelation between carboxylate oxygen and Ln^3+^

**Figure 5:**
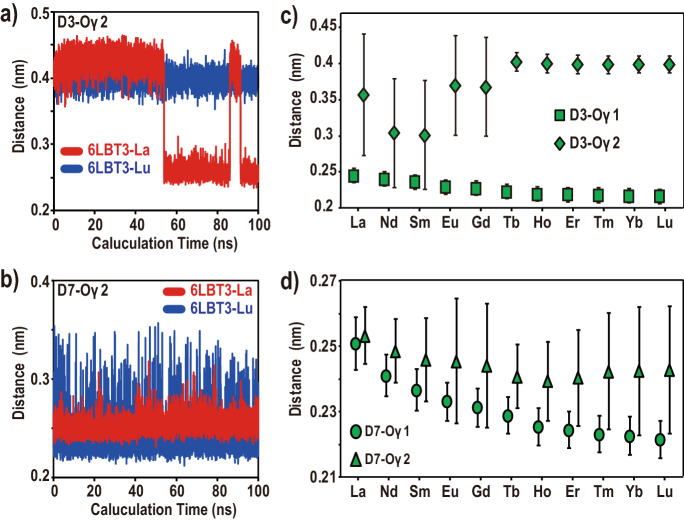
The orientation change of D3 and D7 carboxylate oxygens. Changes in distances between Ln^3+^ and the coordinated carboxylate oxygens of (**a**) D3-Oγ2 and (**b**) D7-Oγ2, observed during 100 ns of MD simulation. The distances between Ln^3+^ and the coordinated carboxylate oxygens of (**c**) D3 and d) D7, compared with the Ln^3+^ species. The symbols indicate average values and the error bars indicate the standard deviation. Several (5000) structures of each 6LBT3-Ln complex obtained by MD simulation at 300 K were used for calculation.

In addition to N5-Oδ1, D3 and D7 carboxylate groups showed a difference in recognition between La^3+^ and Lu^3+^ (Fig. 5a, b, Supplemental Fig. S9c,d). Although all carboxylate groups remained bound to Ln^3+^ throughout the 100 ns calculations under all temperature conditions, D3 and D7 carboxylate oxygens frequently showed bidentate chelation in the 6LBT3-La system, whereas monodentate chelation of these coordinates was observed for 6LBT3-Lu. In the case of monodentate binding, the carboxylate oxygens bind with Ln^3+^ and a nearby water molecule. It is considered that this observation relates to ion size. To confirm this hypothesis, additional MD simulations were performed; the La^3+^ in the 6LBT3-La complex was replaced with Ln^3+^. The results showed that the distance between D3-Oγ1 and Ln^3+^ is shortened as the ionic radii decrease. In contrast, the distance between D3-Oγ2 and Ln^3+^ is shortened from La^3+^ to Sm^3+^, increased from Sm^3+^ to Tb^3+^, and finally becomes insignificant from Tb^3+^ to Lu^3+^ (Fig. 5c). The D7 carboxylate also showed that D7-Oγ1 is simply nearing Ln^3+^ as the ionic radii decrease (Fig. 5d), while the average distance between D7-Oγ2 and Ln^3+^ is shortened from La^3+^ to Tb^3+^, and becomes insignificant from Tb^3+^ to Lu^3+^. In addition, the large error bars observed in Eu^3+^ to Lu^3+^ indicate that D7-Oγ2 easily dissociates from Ln^3+^ as the ionic radii decrease. These observations indicate that the ion size affects the orientation of the coordinated carboxylate oxygens.

### Direct water molecule coordination to Ln^3+^

**Figure 6:**
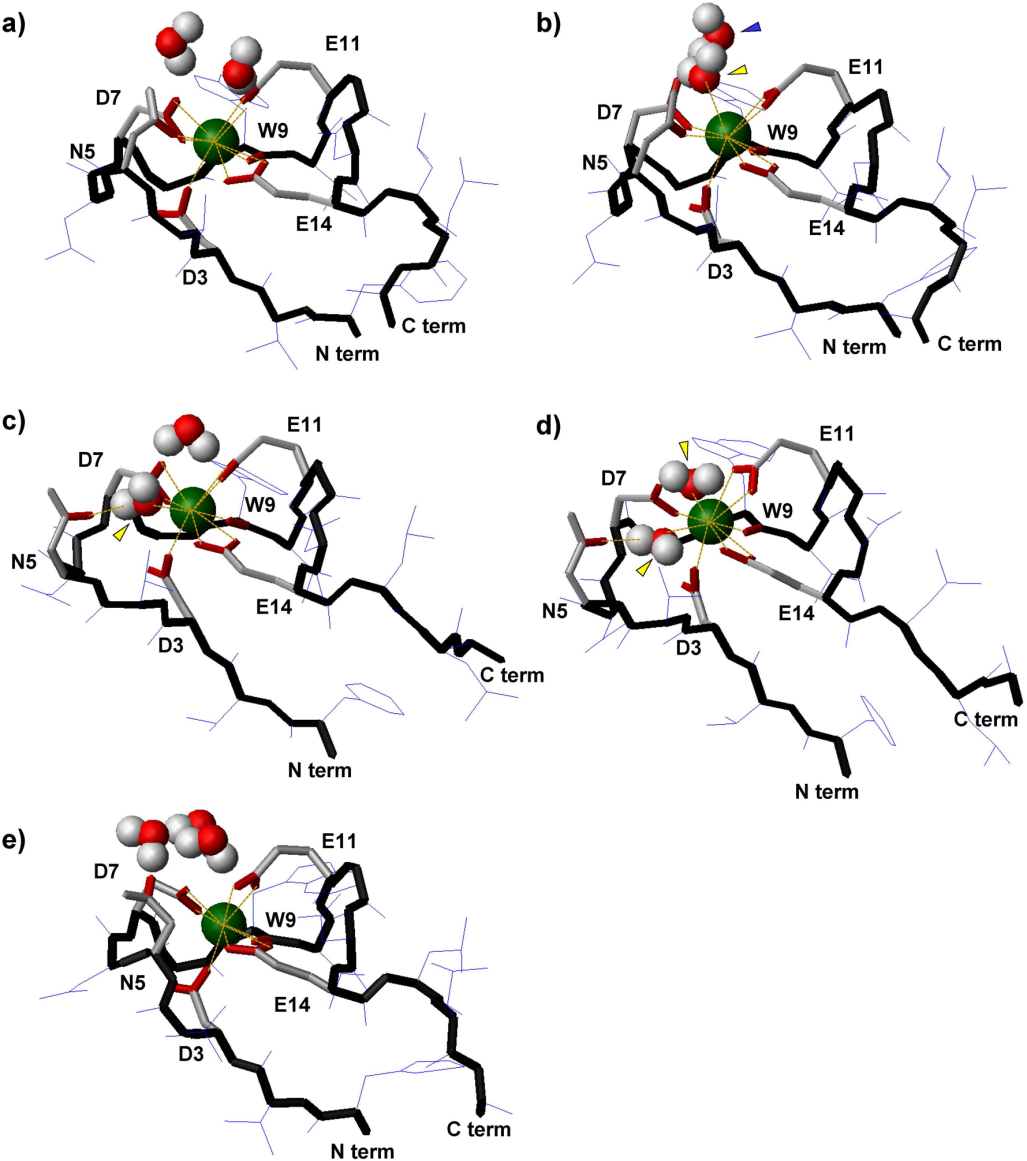
Coordination patterns of LBT3-Ln observed in MD simulations. (**a**) Ln^3+^ bound to six LBT3 residues only. (**b**) Ln^3+^ bound to six LBT3 residues and a water molecule. (**c**) Ln^3+^ bound to five LBT3 residues (excluding N5-Oδ1) and a water molecule. (**d**) Ln^3+^ bound to five LBT3 residues and two water molecules. (**e**) Ln^3+^ bound to five LBT3 residues only. Ln^3+^ are indicated by dark green spheres and water molecules are indicated by red and white spheres (oxygen and hydrogen, respectively). Peptide backbones are shown by solid black lines. Red lines indicate oxygen. All hydrogen molecules of the peptide were omitted for clarity. Yellow arrows indicate the water molecule that is bound to Ln^3+^, while the blue arrow indicates the water molecule that is bound to D7-Oγ2.

To evaluate the origin of the N5-Oδ1 coordination/dissociation, we closely analyzed the local structural changes around Ln^3+^. The coordination patterns of the 6LBT3-La and 6LBT3-Lu systems differed in their interaction with water molecules. Specifically, the 6LBT3-La systems exhibited four coordination patterns: (i) La^3+^ bound only to six LBT3 residues (Fig. 6a); (ii) La^3+^ bound directly to a water molecule in addition to six LBT3 residues (Fig. 6b); (iii) La^3+^ coordinated to five LBT3 residues (excluding N5-Oδ1) and a water molecule (Fig. 6c); and (iv) La^3+^ coordinated to five LBT3 residues (excluding N5-Oδ1) and two water molecules (Fig. 6d). During the 100 ns simulations at various temperatures, the (i) and (ii) states were frequently observed, and the coordination pattern changed as follows: (i) ⇄ (ii) ⇄ (iii) ⇄ (iv). Notably, the coordinated water molecule in (iii) was stabilized by hydrogen bonding with N5-Oδ1 (Fig. 6c). This water molecule invaded the packed complex structure, which led to the coordination of an additional water molecule to La^3+^, resulting in the (iv) state. Moreover, the α helical c-terminus observed in the (i) and (ii) state formed a stretched structure in the (iii) and (iv) state. The 6LBT3-Lu system, on the other hand, showed three types of coordination: Forms analogous to (i) and (iii) above, as well as a unique form in which (v) Lu^3+^ was coordinated with five residues (excluding N5-Oδ1), without a coordinated water molecule (Fig. 6e). The (ii) and (iv) forms were not observed for 6LBT3-Lu. The (i) form was highly stable; coordination changes were only observed above 345 K and followed the path (i) ⇄ (v) ⇄ (iii). The radial distribution functions (RDFs) between water and Ln^3+^ also indicated that the mechanistic differences between the two complexes originated at the point at which water coordination occurred. Direct coordination of water was observed under all conditions tested for 6LBT3-La, whereas it was only observed at 345 K for 6LBT3-Lu (Supplemental Fig. S10a,b). In addition, to check the initial structural effects, La^3+^ and Lu^3+^ were exchanged with each other, and MD simulations were performed at 300 K and 315 K. This showed that direct coordination of water was only observed in the 6LBT3-La complex, even when La^3+^ and Lu^3+^ were exchanged in the initial structures of the simulation (Supplemental Fig. S10c,d). These observations suggest that the direct water coordination strongly depends on the properties of the individual Ln^3+^, and should be related to the difference in size between the ions, rather than being an artifact derived from the initial structural differences.

**Figure 7:**
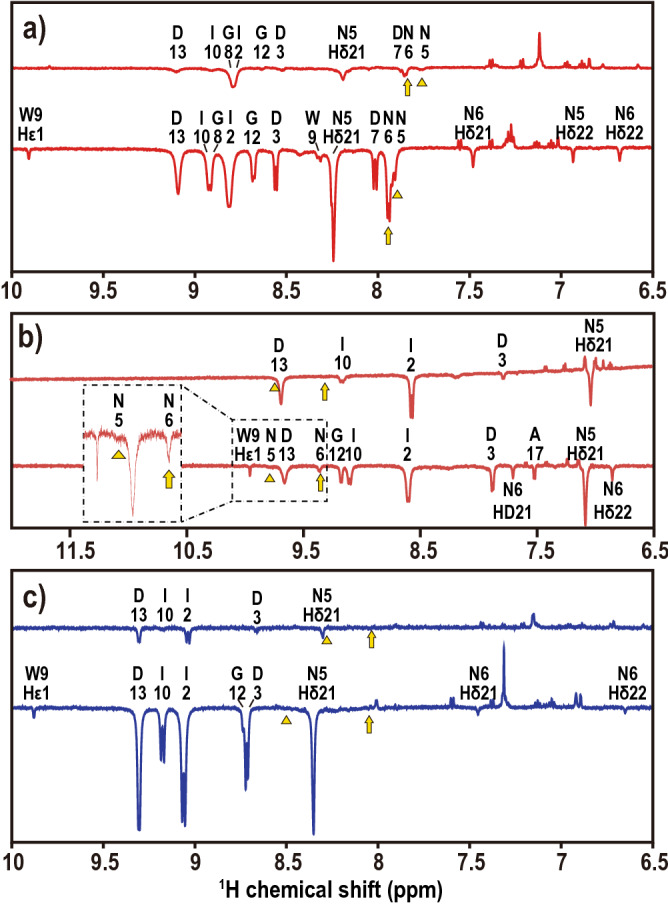
Proton exchange observed in LBT3-Ln complexes using NMR spectroscopy. (**a**) LBT3-La, (**b**) LBT3-Sm, and (**c**) LBT3-Lu were analyzed using CLEANEX-PM. The experiments were performed at 10 °C (upper spectrum) and 25 °C (lower spectrum). The assignments indicated in each panel demonstrate the occurrence of proton exchange. The amide protons are represented with a single letter abbreviation and number. The triangle and arrow symbols indicate the positions of the N5-HN, N6-HN peaks, respectively.

NMR spectroscopy also supported the simulated results described above. Proton exchange was observed for N5-HN and N6-HN in the LBT3-La complex at 10 °C and 25 °C (Fig. 7a). The LBT3-Sm complex also showed proton exchange for N5-HN and N6-HN at 25 °C (Fig. 7b), but no amide proton exchange was observed for the LBT3-Lu complex (Fig. 7c). Proton exchange reflects the degree of solvent accessibility and mobility of each site. Considering the structures, water molecules are thought to exist inside the LBT3-La complex. These results also agreed well with the ^1^H NMR spectral differences between LBT3-La and LBT3-Lu; N5-HN of LBT3-La exhibited a comparably large shift toward the higher magnetic field, 0.5 ppm, compared with LBT3-Lu (Supplemental Fig. S2). To confirm that the largest chemical shift observed for G8-HN is also induced by water molecule interaction, the RDFs between water and G8-HN were analyzed. A clear difference was observed, namely that a water molecule locates closer to the G8-NH of LBT3-La than to that of LBT3-Lu (Supplemental Fig. S11a,b). The detailed structural analysis showed that the difference originates from the orientation of the D3 carboxylate oxygen. When the D3 carboxylate exhibits bidentate chelation, a water molecule draws close to G8-HN (Supplemental Fig. S11c). In the case of monodentate chelation, the freed carboxylate oxygen (D3-Oγ2) traps a water molecule, preventing the water molecule from drawing close to G8-HN (Supplemental Fig. S11d). These experimental and computational results demonstrate that there is a notable difference between the interaction of water molecules with LBT3-La and LBT3-Lu. In particular, the water infiltration easily occurs in the case of the La^3+^ complexation, whereas this is a rare event in the Lu^3+^ recognition.

### Effect of N5-Oδ1 dissociation to the complex structure

To elucidate the effect of N5-Oδ1 dissociation from Ln^3+^, we performed another MD simulation using artificial complex structures, where which Ln^3+^ interacts with the five sites (excluding N5-Oδ1) of LBT3. The initial structures were constructed using the program CYANA based on the NOESY spectra; the five residues were set as binding sites. The obtained structures, which were named 5LBT3-La and 5LBT3-Lu, showed high similarity with a backbone rmsd value of 0.456 Å (Supplemental Fig. S12). Using these structures, we performed MD simulations under the same conditions as for 6LBT3-Ln. Although N5-Oδ1 did not bind with Ln^3+^ at any point during the calculation (Supplemental Fig. S13a,b), the other coordination sites maintained their binding with Ln^3+^, similarly to the 6LBT3-Ln system (Supplemental Fig. S13c,d). In the 5LBT3-La system, as expected, direct water coordination was also frequently observed (Supplemental Fig. 14a). Structure (iii), which includes direct coordination of one water molecule to La^3+^, was the most frequently observed throughout the calculation at all the tested temperatures. The (iii) ⇄ (iv) structural transition was occasionally observed. By contrast, in the 5LBT3-Lu system, direct water coordination was only observed at higher temperatures (≥ 315 K) (Supplemental Fig. 14b). Only structure (v), which does not include direct coordination of any water molecule to Lu^3+^, was observed at low temperatures (≤ 300 K), and the coordination transition (v) ⇄ (iii) was observed at higher temperatures (≥ 315 K). Despite the artificial constraints in the initial structures of 5LBT3-Ln, these results indicate that when N5-Oδ1 is in the dissociation state, La^3+^ is exposed in the solvent, whereas Lu^3+^ is highly covered in LBT3.

## Discussion

The overall binding pattern between LBT3 and Ln^3+^ is as follows. In aqueous solution, free LBT3 and Ln^3+^ are hydrated or coordinated, respectively, by water molecules that are released to the bulk solvent upon complex formation^21,22^. LBT3 surrounds the Ln^3+^ by using its six residues and makes a partially α helical compact structure. According to the experimental and computational results, the structural factor underlying the large difference in binding affinity of LBT3 with each Ln^3+^ can be explained as follows. From Sm^3+^ to La^3+^, as the coordination sphere enlarges, water molecules can directly interact with Ln^3+^ with ease. Because the coordinated water molecule is located next to N5-Oδ1, which is the most weakly coordinated among the ligands, it interposes itself between N5-Oδ1 and Ln^3+^. In addition, this water molecule invasion allows another water molecule to coordinate with Ln^3+^ directly; these water molecules interfere with the rebinding of N5-Oδ1 to Ln^3+^. The dissociated N5-Oδ1 results in the structural fluctuations of the N5 sidechain, and this local fluctuation spread may result in the structural disruption of the LBT3-Ln complex. In other words, the complexes with water molecules directly bound to Ln^3+^ are structurally flexible, resulting in a reduction in binding affinities. By contrast, from Sm^3+^ to Lu^3+^, the ion sizes are thought to be small enough to be completely covered by LBT3. Here, direct interaction with water is rare, resulting in stable complexation and high affinity.

The thermodynamic analysis indicates the magnitude of energies of various reactions during complexation. The water release event is enthalpically unfavorable, especially for such a large trivalent ion, and is not compensated by the enthalpically favorable reactions such as electrostatic bond formation between LBT3 and Ln^3+^, which results in a stable 8 or 9 coordination structure, and intramolecular hydrogen bond formation. Moreover, complex formation decreases the entropy of the peptide chain, which also disfavors binding. However, the release of water is very entropically favorable and represents the driving force of the reaction, ultimately overcoming any thermodynamically unfavorable effects. Although it is difficult to isolate various reactions and these energies, the structural features and thermodynamic parameters of each of the LBT3-Ln complexes allow us to interpret the recognition specificity as follows.

**Figure 8:**
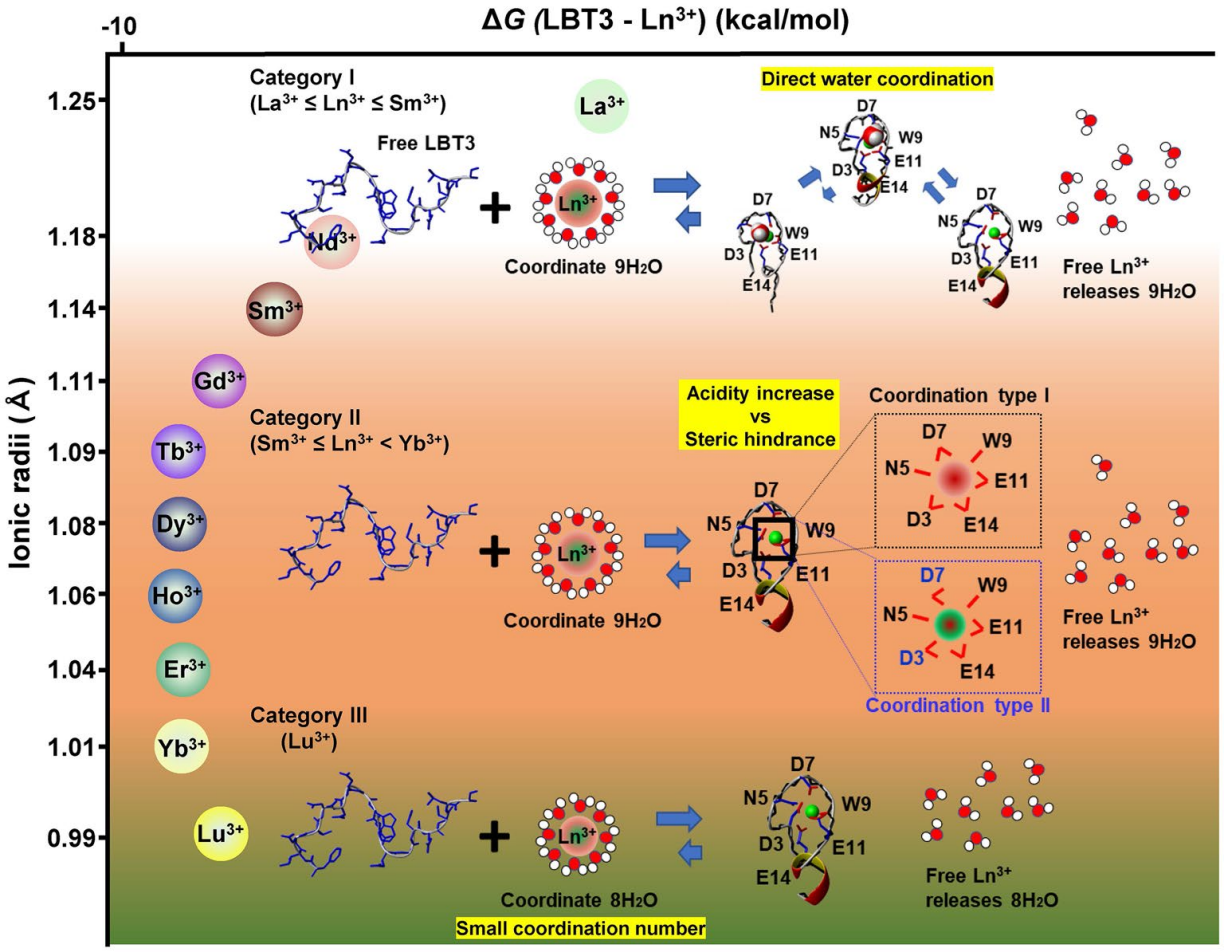
Schematic illustration of the binding of LBT3 with Ln^3+^. The binding of LBT3 with Ln^3+^ is categorized into three: La^3+^ ≤ Ln^3+^ ≤ Sm^3+^, Sm^3+^ ≤ Ln^3+^ ≤ Yb^3+^, and Lu^3+^. The second category is further divided into two subclasses, Sm^3+^ ≤ Ln^3+^ < Tb^3+^ and Tb^3+^ ≤ Ln^3+^ ≤ Yb^3+^. The graphics and yellow highlighted sentences in each category indicate the characteristic factors.

Considering the Δ*H*, Δ*S* (Supplemental Table S1) and structural features, the binding of LBT3 with Ln^3+^ could be divided into three: the first is La^3+^ ≤ Ln^3+^ ≤ Sm^3+^ (‘≤’ indicates the largeness of the atomic number), the second is Sm^3+^ ≤ Ln^3+^ ≤ Yb^3+^, and third is Lu^3+^ (Fig. 8). From La^3+^ to Sm^3+^ (top of Fig. 8), the complexes are less stable than the other classes. NMR and MD simulation indicated that the water molecule not only interacts with Ln^3+^ directly, but is also able to access N5-HN, especially in the LBT3-La complex. Considering that the structure constructed by NMR reflects the average structure, a non-negligible amount of the (ii) and (iii) types of complexation mode could co-exist with the (i) form in the case of the LBT3-La complex. Considering this, the comparably large Δ*H* in La^3+^ (3.83 kcal/mol) might reflect the dissociation of N5-Oδ1 from Ln^3+^ and the reduced intramolecular bond formation. As the ion size decreases from La^3+^ to Sm^3+^, the direct coordination of water molecules becomes less, and the LBT3 ligands are pulled tightly to Ln^3+^ because of the increase in acidity. As a result, the enthalpically disfavoring N5-Oδ1 dissociation becomes less, and the intramolecular bond formation is induced, resulting in a decrease in Δ*H* (ΔΔ*H*_Sm-La_ = -2.2 kcal/mol). Although the reactions for the stabilizing complex are entropically disfavored, the costs are small (-TΔΔ*S*_Sm-La_ = 0.37 kcal/mol), resulting in an affinity increase (ΔΔ*G*_Sm-La_ = -1.83 kcal/mol).

Sm^3+^ ≤ Ln^3+^ ≤ Yb^3+^ can be further divided into two subclasses: Sm^3+^ ≤ Ln^3+^  < Tb^3+^ and Tb^3+^ ≤ Ln^3+^ ≤ Yb^3+^ (middle of Fig. 8). In both classes, as the ion sizes are small enough to be completely covered by LBT3, notable complex structural alteration (such as direct water coordination with Ln^3+^) is negligible. Most of the complex is thought to construct the (i) type of complexation. However, the affinity of the former class increases (ΔΔ*G*_Tb-Sm_ = − 0.54 kcal/mol), while that of the latter class does not change (ΔΔ*G*_Yb-Tb_ = − 0.01 kcal/mol). The thermodynamic parameters indicate that the enthalpically favorable factor is largely affected in the former class rather than the latter (ΔΔ*H*_Tb-Sm_ = 0.43 kcal/mol, − TΔΔ*S*_Tb-Sm_ = − 0.97 kcal/mol, ΔΔ*H*_Yb-Tb_ = 0.85 kcal/mol, − TΔΔ*S*_Yb-Tb_ = − 0.86 kcal/mol). Because the structures in both subclasses are structurally stable, the Δ*H* difference should originate in the bond formation between LBT3 and Ln^3+^. Therefore, the phenomenon whereby acidity increases as the atomic number increases is considered to be the main reason for the affinity increase in Sm^3+^ ≤ Ln^3+^  < Tb^3+^. These results also mean that an unknown effect compensates for the effect of the acidity increase, resulting in a nearly constant affinity from Tb^3+^ to Yb^3+^. This effect was related to steric hindrance. The Ln^3+^ with a smaller ionic radius more strongly attracts the coordination atoms, although it also simultaneously induces closer coordination, which should result in repulsion. The intricately folded peptide structure might be distorted by the strong attraction. It was considered that the observed orientation change of the coordinated carboxylates of D3 and D7 results from the compensation of these steric hindrances.

Regarding Lu^3+^ (bottom of Fig. 8), the LBT3-Lu complex is also occupied by the (i) form. Because of its small ion size, Lu^3+^ is known to coordinate with notably fewer water molecules among the series of Ln^3+^^1^. Therefore, when LBT3 binds with Lu^3+^, it needs less energy for the de-coordination of water molecules than Yb^3+^. This also means that Lu^3+^ releases fewer water molecules than Yb^3+^. The decrease in Δ*H* (ΔΔ*H*_Lu-Yb_ = − 0.4 kcal/mol) and Δ*S* (TΔΔ*S*_Lu-Yb_ = − 0.63 kcal/mol) is predicted to reflect this character.

In conclusion, this is the first report that precisely explains how a flexible linear peptide LBT3 recognizes a Ln^3+^ species. Our findings clearly indicate that water molecules play important roles, both in the reaction as a whole, and in the recognition specificity. Previously, the contributions of water molecules in protein-target interactions have been well studied^23,24^. For example, in the potassium ion channel, which recognizes the difference between potassium and sodium ions, the protein structure controls the coordination/dissociation of water molecules around the ions. This is the key factor in distinguishing between these two ions, whose radii differ by less than 0.5 Å^25,26^. Interestingly, a highly stabilized membrane protein and a flexible peptide uses water molecules to distinguish sub-angstrom size differences. This finding could be important for the design of artificial molecules that considers solvent molecules as a part of the target molecule rather than solely as a solvent.

## Experimental procedures

### Materials

All lanthanide nitrates were purchased from Sigma-Aldrich (St. Louis, MO). MES-*d*_13_ was purchased from Cambridge Isotope Laboratories Inc. (Tewksbury, MA).

### ITC analysis

The thermodynamic parameters of binding between Ln^3+^ and the peptides were analyzed at 10 °C by isothermal titration calorimetry (ITC, MicroCal VP-ITC, MicroCal Worcestershire, UK). The synthetic peptides and lanthanide nitrate (Ln(NO_3_)_3_) were each dissolved in 50 mM MES buffer containing 100 mM NaCl (pH 6.0). The experimental conditions were adjusted according to the manufacturer’s instructions. Typically, LBT3 solution (15 µM) was placed in the calorimeter cell, and then Ln(NO_3_)_3_ (300 µM) was loaded into the syringe injector_._ The experiments included 20 injections, whereby an initial 2 µL injection was used to account for dilution of the syringe, and the remaining injections were 10 µL with a 300 s delay between each injection. The effect of Ln^3+^ dilution in the cell was calculated by subtraction of titration data for a blank, which consisted of titrating Ln^3+^ into a buffer solution. Each binding parameter, including stoichiometry (*N*), association constant (*K*), and binding enthalpy (Δ*H*), was calculated using the ITC Origin Analysis Software version 7.0 (Malvern). For the samples that did not show a sigmoidal response owing to a low *K* value, the thermodynamic parameters were calculated by fixing the stoichiometry to 1.0 according to the manufacturer’s instructions.

### NMR spectroscopy

Nuclear magnetic resonance (NMR) experiments were carried out using a Bruker Avance spectrometer equipped with a TCI cryoprobe. Standard 5 mm NMR tubes were used for the measurements. The samples were prepared in 30 mM MES-d_13_ buffer (H_2_O/D_2_O = 90:10, pH 6.0, Sigma-Aldrich). To equilibrate the binding reaction, all measurements were performed at least 10 min after mixing. All assignments were carried out using a combination of total correlation spectroscopy (TOCSY), nuclear Overhauser effect spectroscopy (NOESY), heteronuclear single-quantum correlation spectroscopy (HSQC)^27^. In addition, CLEAN chemical exchange spectroscopy (CLEANEX-PM) was carried out to examine the solvent-accessible residues^28^. TOCSY was conducted with a mixing time of 80 ms, NOESY with a mixing time of 400 ms, and CLEANEX-PM with a mixing time of 200 ms. Titration experiments were performed by adding Ln^3+^ solutions to the peptide solutions. ^1^H and TOCSY spectra were measured at each titration step to trace the chemical shift changes. All NMR data were processed and analyzed with Topspin 3.1, NMRPipe^29^, and Sparky^30^.

### Structural calculation

The peptide structures were calculated using the program CYANA 2.1 for the automatic assignment of the NOE peak lists. The upper limits of the distance restraints were calculated from the NOESY cross-peak intensities using the calibration routine of CYANA. For calculation of the 6LBT3-Ln complex structures, the coordination oxygen atoms, the upper and lower limit distance restraints between Ln^3+^ to coordinated oxygen atoms were set according to the crystal structure of LBTv-Tb; D3-Oγ1, N5-Oδ1, D7-Oγ1, W9O, E11-Oε1, E11-Oε2, E14-Oε1, and E14-Oε2 were set as coordination sites; the upper limit was set to 3.0 Å, and the lower limit was set to 2.1 Å^11^. For calculation of the 5LBT3-Ln complex structures, D3-Oγ1, D7-Oγ1, W9O, E11-Oε1, E11-Oε2, E14-Oε1, and E14-Oε2 were set as coordination sites. The structure with the lowest target function was used in subsequent MD simulations.

For the structural refinement, GROMACS software was used. Briefly, the 20 structures obtained by CYANA structural calculation were treated with the same procedure as subsequent MD simulation analyses until the production run. After 100 ps constant pressure equilibration, MD simulations with distance restraints were performed at 500 K for 10,000 steps. The structures were then minimized at the end of calculations. The structural representation was performed by MOLMOL 1.07 software^31^.

### Molecular Dynamics Simulations and Analysis

All MD simulations were performed using GROMACS software^32^. Each LBT3-Ln complex was solvated in a square box of 2589 water molecules. All Glu and Asp residues were set as negatively charged forms, whereas the other residues were set to neutral. As a result, the net charge of the LBT3-Ln complex became -2, and Na^+^ counterions were added to neutralize the net charge. CHARMM36 force fields were employed for the peptide model^33^, TIP3P was used for water molecules, and the model of Migliorati et al.^34^ was used for the Ln^3+^. The long-range electrostatic interactions were treated with particle-mesh Ewald summation^35^, and a 1.2 nm cutoff was used for Lennard–Jones and Coulombic interactions. All bonds between hydrogen and heavy atoms were constrained using the LINCS algorithm^36^.

In the simulations, we initially performed 50,000 steps of minimizations with a restricted LBT3-Ln complex and then equilibrated the minimized systems following 100 ps constant pressure equilibrations. After equilibration, an additional 100 ps constant-volume equilibration and 100 ps constant pressure equilibration were performed without restrictions. The following production runs were then carried out for 100 ns. The temperature was kept constant using the stochastic velocity rescaling method^37^, and the pressure was kept constant at 1 atm using the Parrinello-Rahman method^38^. A 2 fs time step was employed in all simulations. Other simulation setups were followed according to the manufacturer’s instructions and the main text of our paper.

## Supplementary information

Supplementary Information.

## Data Availability

The assigned ^1^H and ^13^C chemical shifts of LBT3-Ln have been deposited in the BMRB (36356, 36357) and structural coordinates have been deposited in the PDB (7CCN, 7CCO). All data are contained within the manuscript.
