# Supplementary material for: The origins of binding specificity of a lanthanide ion binding peptide
Source: Sci Rep. 2020 Nov 10;10:19468. doi: 10.1038/s41598-020-76527-y (PMC7656248; doi:10.1038/s41598-020-76527-y)
Supplement: Supplementary file 1 — Supplementary Information. [file 41598_2020_76527_MOESM1_ESM.pdf]

The origins of binding specificity of a lanthanide ion binding peptide

Takaaki Hatanaka,*† Nobuaki Kikkawa,† Akimasa Matsugami,‡ Yoichi Hosokawa,† Fumiaki Hayashi, *‡ Nobuhiro Ishida†

†Toyota Central R&D Labs., Inc., 41-1, Nagakute, 480-1192 Aichi, Japan

‡Advanced NMR Application and Platform Team, NMR Research and Collaboration Group, NMR Science and Development Division, RIKEN SPring-8 Center, 1-7-22 Suehiro-cho, Tsurumi-ku, Yokohama, 230-0045 Kanagawa, Japan

2-15 …………………… Supplemental Figure S1-S14

16 ……………………… Supplemental Table S1

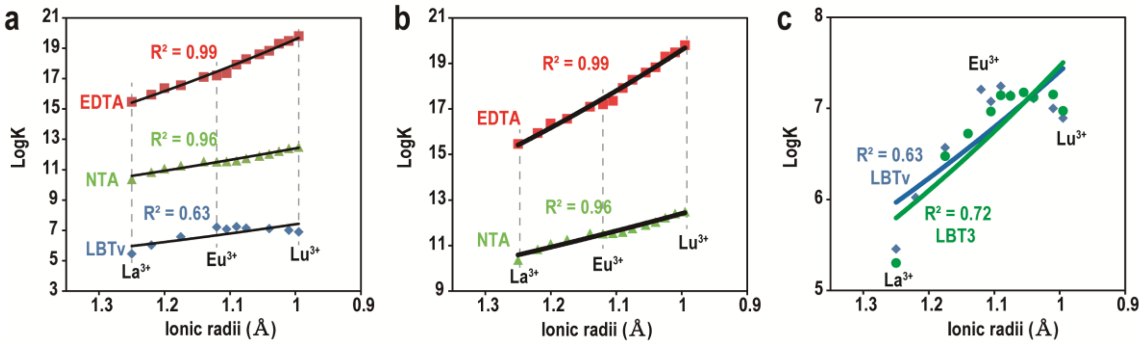

Figure S1. Affinity between Ln^{3+} and its chelating molecules as a function of the ionic radii of Ln^{3+}. K = [Ln^{3+}-chelator]/ [Ln^{3+}][chelator]. The data for EDTA and NTA were obtained from ref. 9, 10 in the main text. The approximate curves indicate the exponential trendline. Panel (b) highlights the EDTA and NTA data in panel (a). Panel (c) highlights the LBTv data in (a) overlaid with our data (main Figure 1). LBT3 is the peptide that was mainly used in this study. LBTv is the one of the LBT variant that is reported in ref. 11. The amino acid sequence of LBT3 is FIDTNNDGWIEGDELLA, and LBTv is YIDTNNDGWYEGDELLA. These results indicate that the affinities of EDTA and NTA to Ln^{3+} are highly correlated to ionic radii but that of the LBT series is not. In other words, the increase in acidity of the Ln^{3+} is the main factor for the increase in binding affinity with EDTA and NTA. On the other hand, the increase in affinity of LBT with Ln^{3+} is not only based on an increase in Ln^{3+} acidity, but includes multiple factors.

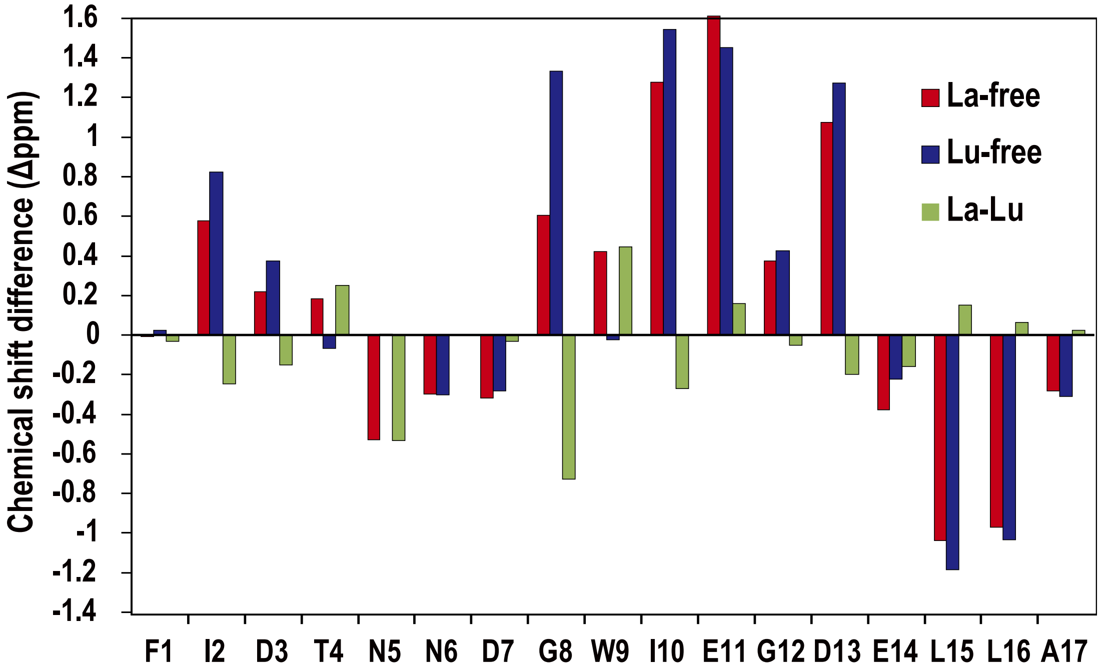

Figure S2. Differences in amide proton chemical shifts. La-free indicates the chemical shift difference between LBT3-La and free LBT3. Lu-Free indicates the chemical shift difference of between LBT3-Lu and free LBT3. La-Lu indicates the chemical shift difference between LBT3-La and LBT3-Lu.

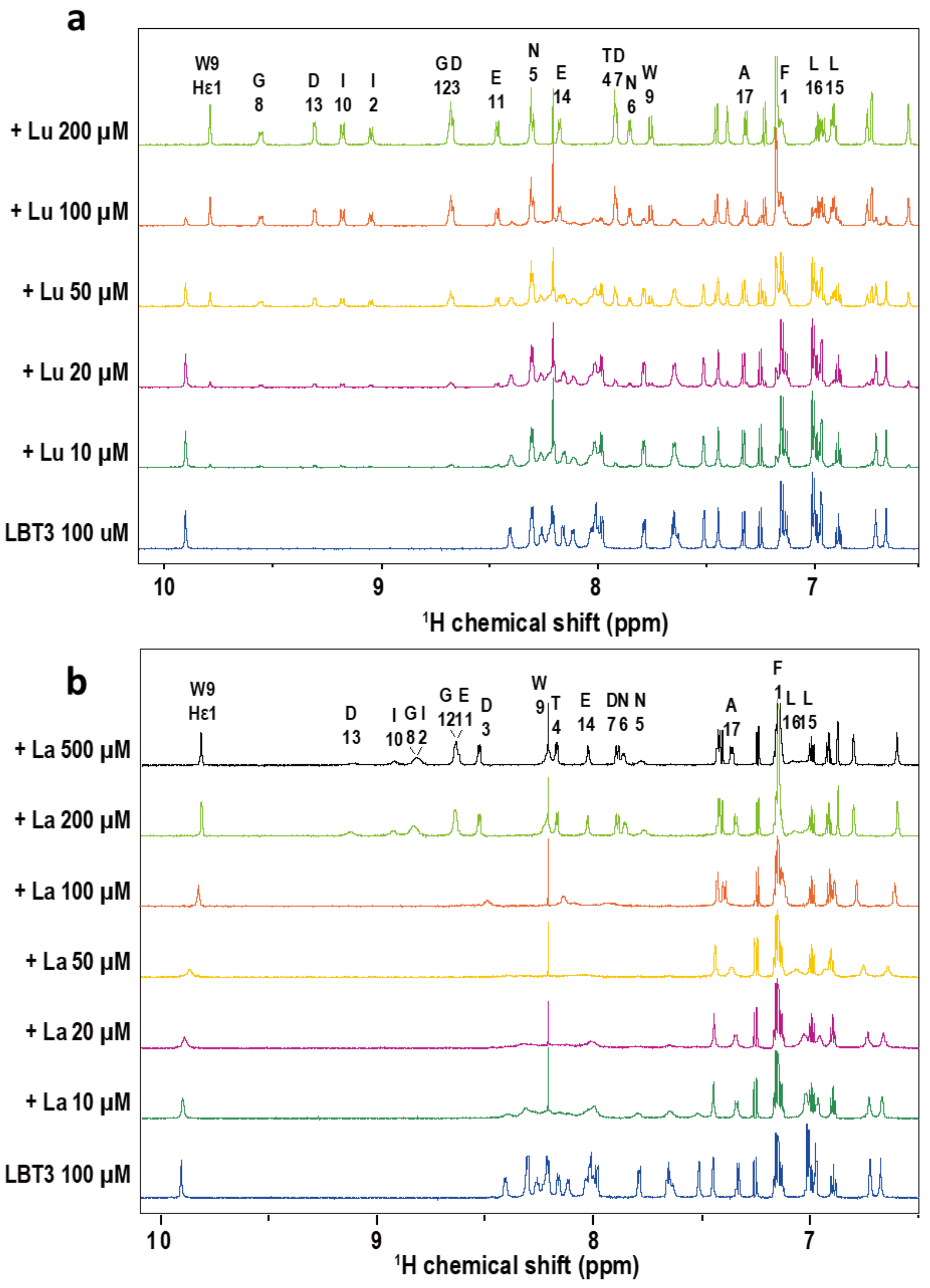

Figure S3. The chemical shift changes in the amide proton region of LBT3 with the addition of (a) Lu^{3+} or (b) La^{3+}. The experiments were performed at 10 °C. Titration of Lu^{3+} induced a gradual increase in the number of chemical shifts derived from the LBT3-Lu complex. Increasing the concentration of La^{3+} to 50 μM resulted in the disappearance of all NH resonances, and chemical shifts derived from the LBT3-La complex appeared at 200 μM La^{3+}. In both cases, chemical shifts reached equilibrium at twice the Ln^{3+} concentration compared to LBT3.

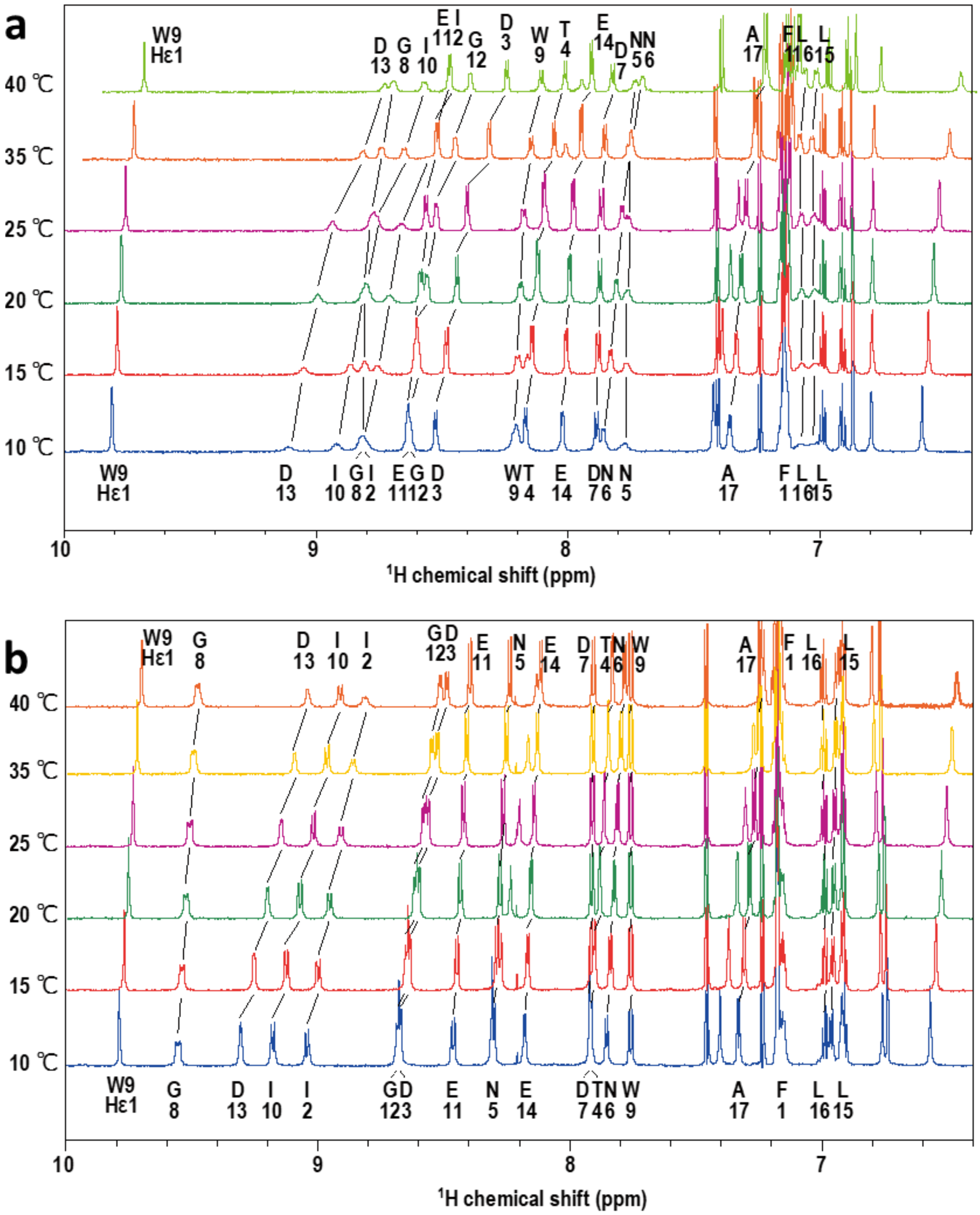

Figure S4. The temperature effect on ^{1}H chemical shift of LBT3-La (a) and LBT3-Lu (b).

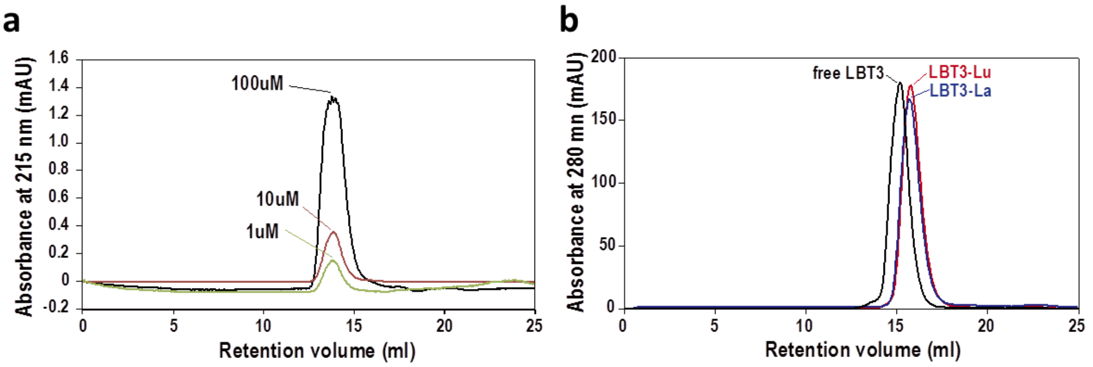

Figure S5. **Gel-filtration chromatography.** (a) Free LBT3 was analyzed at 3 different concentrations: 1, 10, and 100 µM. There was almost no difference in the retention volume of these samples. (b) Free LBT3, LBT3-La, and LBT3-Lu were compared by retention volume. In the case of complex analysis, Ln^{3+} were dissolved in the mobile phase to maintain the complexation state. All experiments were performed at 10 °C. These results indicate that free LBT3 and LBT3-Ln complexes exist as a monomer in solution. This result can be easily understood, as LBT3 contains 5 acidic amino acids and exhibits a charge of -4 at pH 6, which would result in repulsion between the free peptides or complexes.

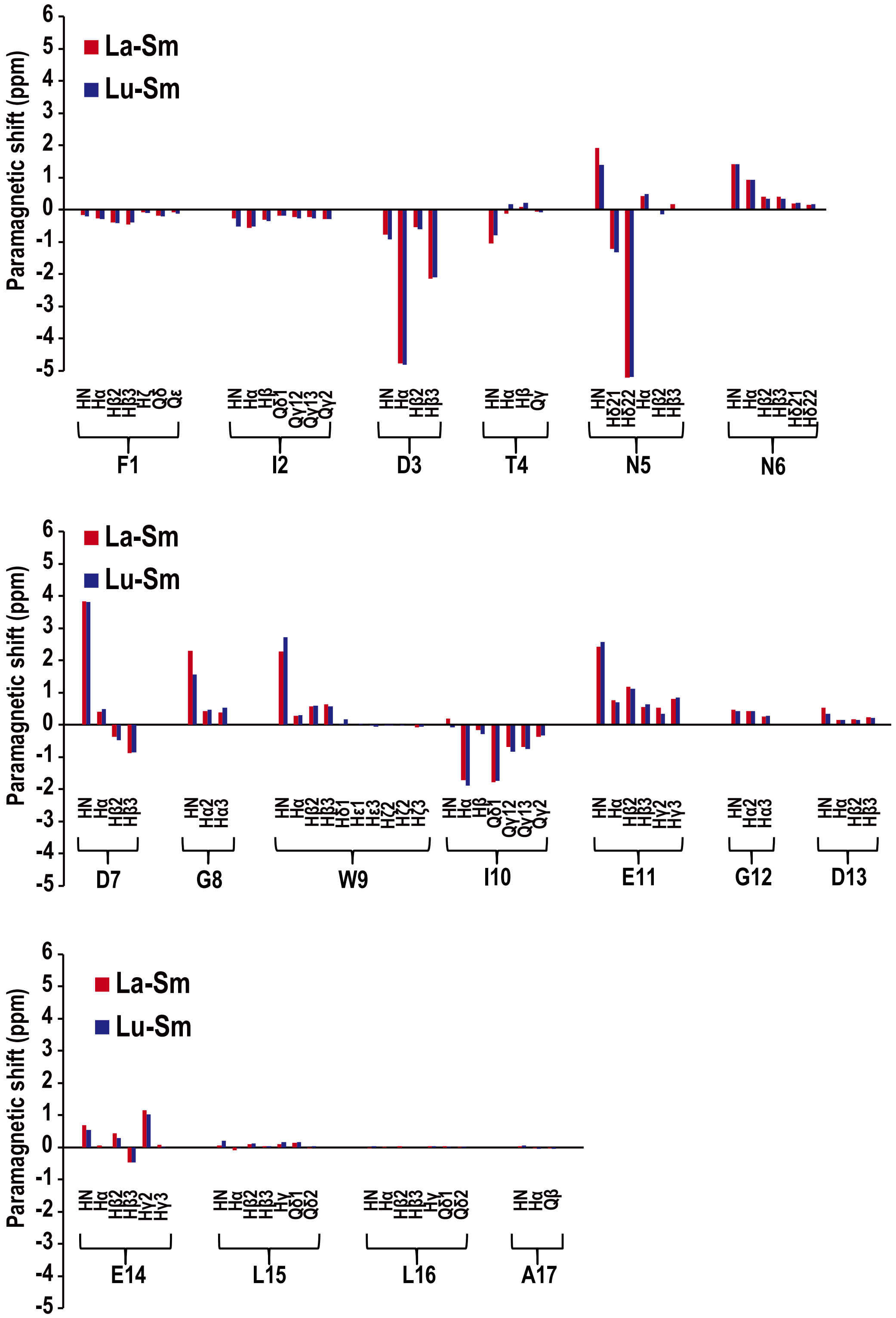

Figure S6. Paramagnetic effect. The difference of chemical shit between LBT3-La/LBT3-Lu and LBT3-Sm were plotted. Positive shifts indicate a downfield shift, while negative shifts indicate an upfield shift.

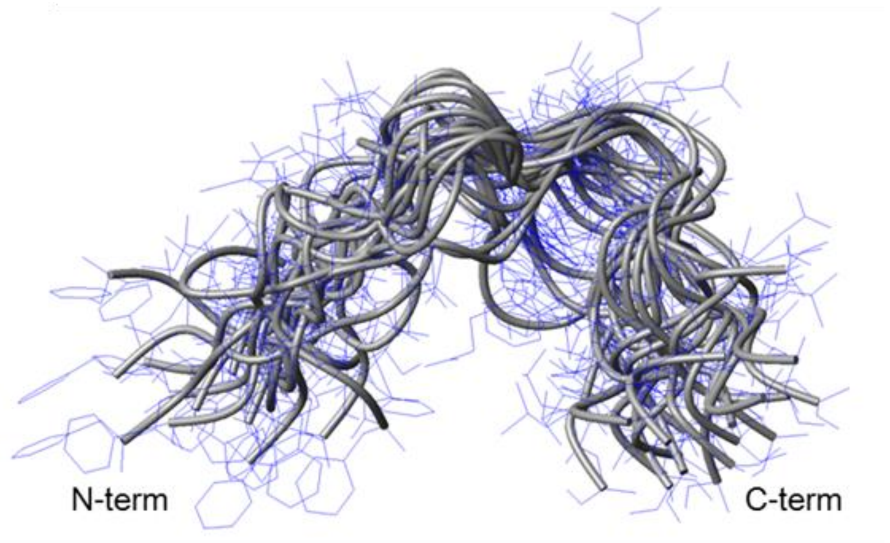

Figure S7. Free LBT3 structure in solution elucidated by NMR. The ensemble of the 20 best structures is shown, and which had a backbone rmsd of 1.82 ± 0.46 Å (residues 4-16) and an average target function of 2.62 ± 0.37. Gray tube: backbone, Blue line: sidechain.

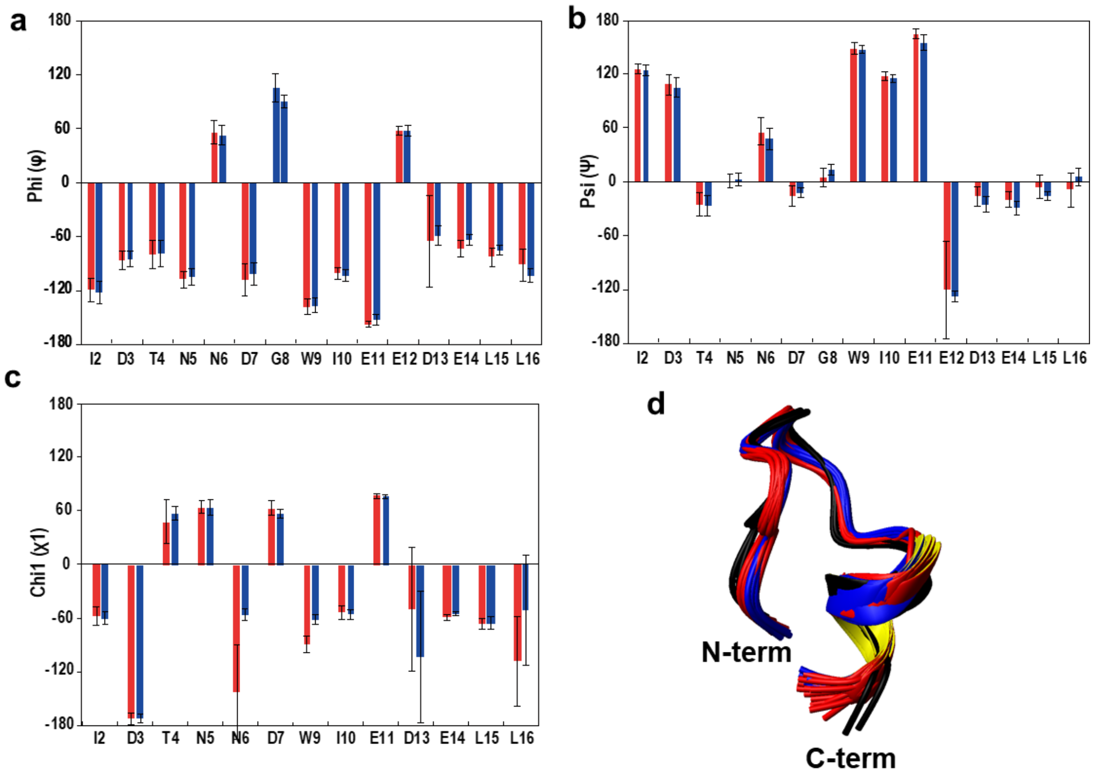

Figure S8. Comparison of 6LBT3-La and 6LBT3-Lu.

Comparison of the (a) Phi, (b) Psi, and (C) Chi1 angles of LBT3-La and LBT3-Lu. (d) Superimposed backbone structures of 6LBT3-La (red ribbon), 6LBT3-Lu (blue ribbon), and LBTv-Tb (black ribbon). The structures of LBTv-Tb were previously evaluated using crystal structure analysis (PDB ID: 1TJB). LBTv has a slightly different amino acid sequence than the LBT3 used in our experiment. LBT3: FIDTNNDGWIEGDELLA. LBTv: YIDTNNDGWYEGDELLA.

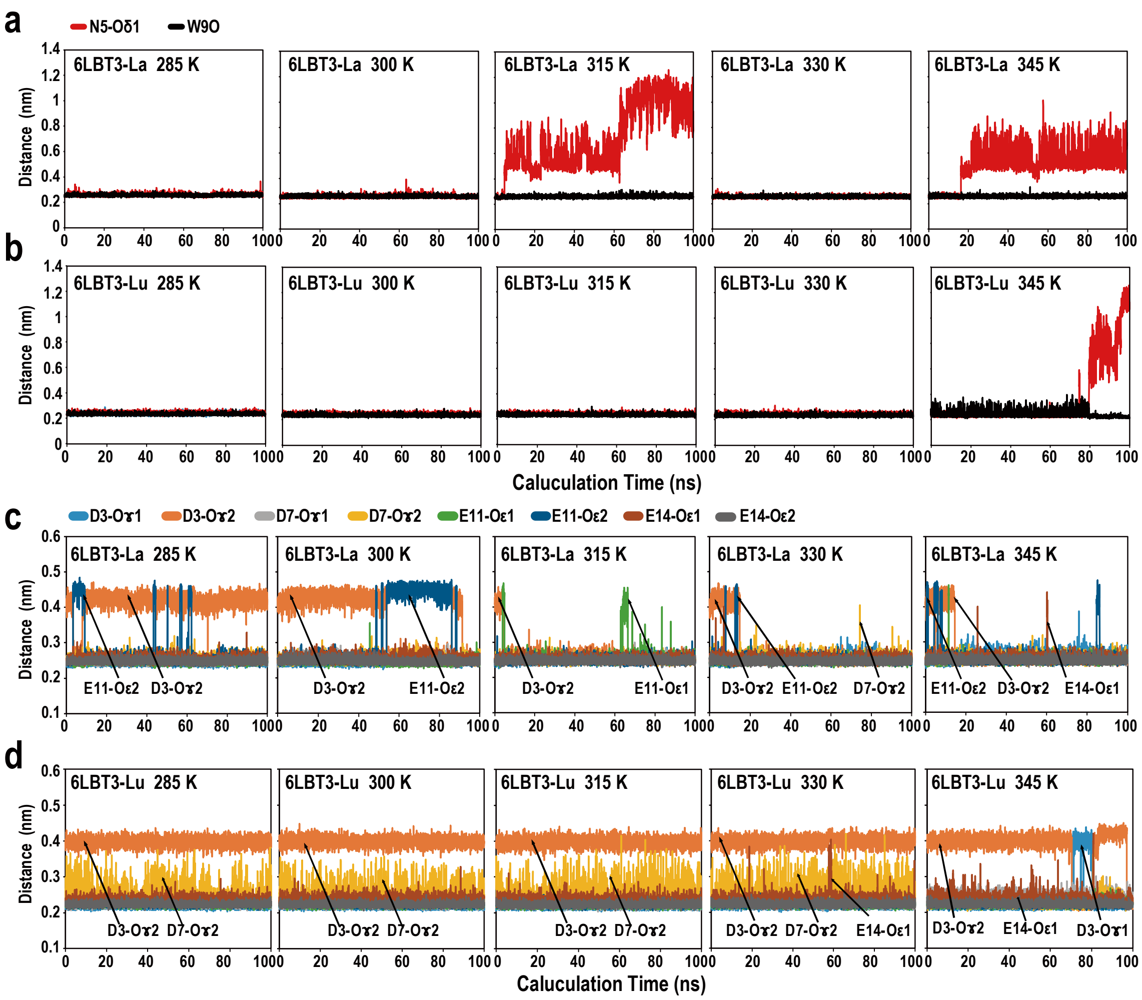

Figure S9. Changes in the distances between Ln^{3+} and coordinated oxygens during MD simulations of the 6LBT3-Ln system. (a,b) Changes in the distances between Ln^{3+} and the coordinated carbonyl oxygens N5-Oδ1 and W9O. (c, d) Changes in the distance between Ln^{3+} and the coordinated carboxylate oxygens D3-Oγ1, D3-Oγ2, D7-Oγ1, D7-Oγ2, E11-Oε1, E11-Oε2, E14-Oε1, and E14-Oε2. (a) and (c) represent the 6LBT3-La system, and (b) and (d) represent the 6LBT3-Lu system. The simulations were conducted at different temperatures (285 K to 345 K), as indicated in each panel. Dissociation of the carboxylate groups was not observed under any conditions. The carboxylate groups maintained bidentate chelation or monodentate chelation with the Ln^{3+} throughout the calculation.

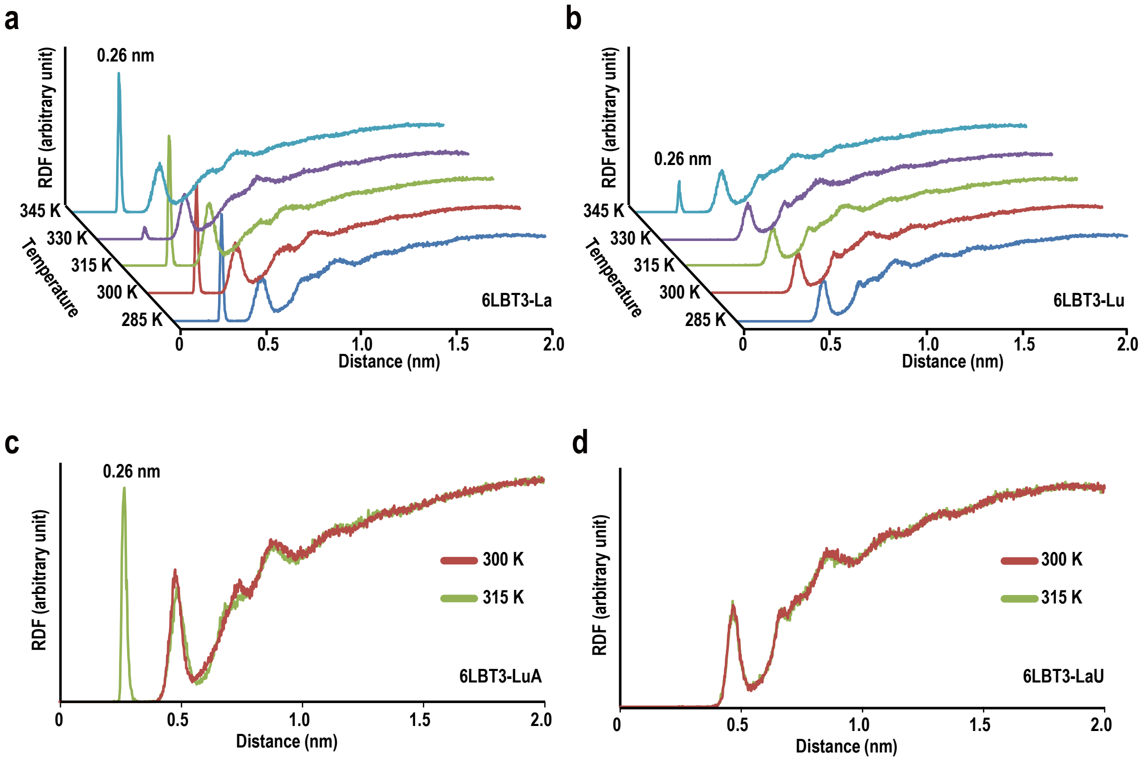

Figure S10. Radial Distribution Functions (RDF) of water molecules around 6LBT3-Ln.

(a) 6LBT3-La. (b) 6LBT3-Lu. (c) La^{3+} in the 6LBT3-La complex was exchanged with Lu^{3+}, which is named 6LBT3-LaU. (d) Lu^{3+} in the 6LBT3-Lu complex was exchanged with La^{3+}, which is named 6LBT3-LuA. The horizontal axis indicates the distance between Ln^{3+} and an oxygen atom of a water molecule. The peak at 0.26 nm indicates the existence of a water molecule that directly coordinates Ln^{3+}. These results indicate that water coordination occurs much easier in the 6LBT3-La complex than in the 6LBT3-Lu complex.

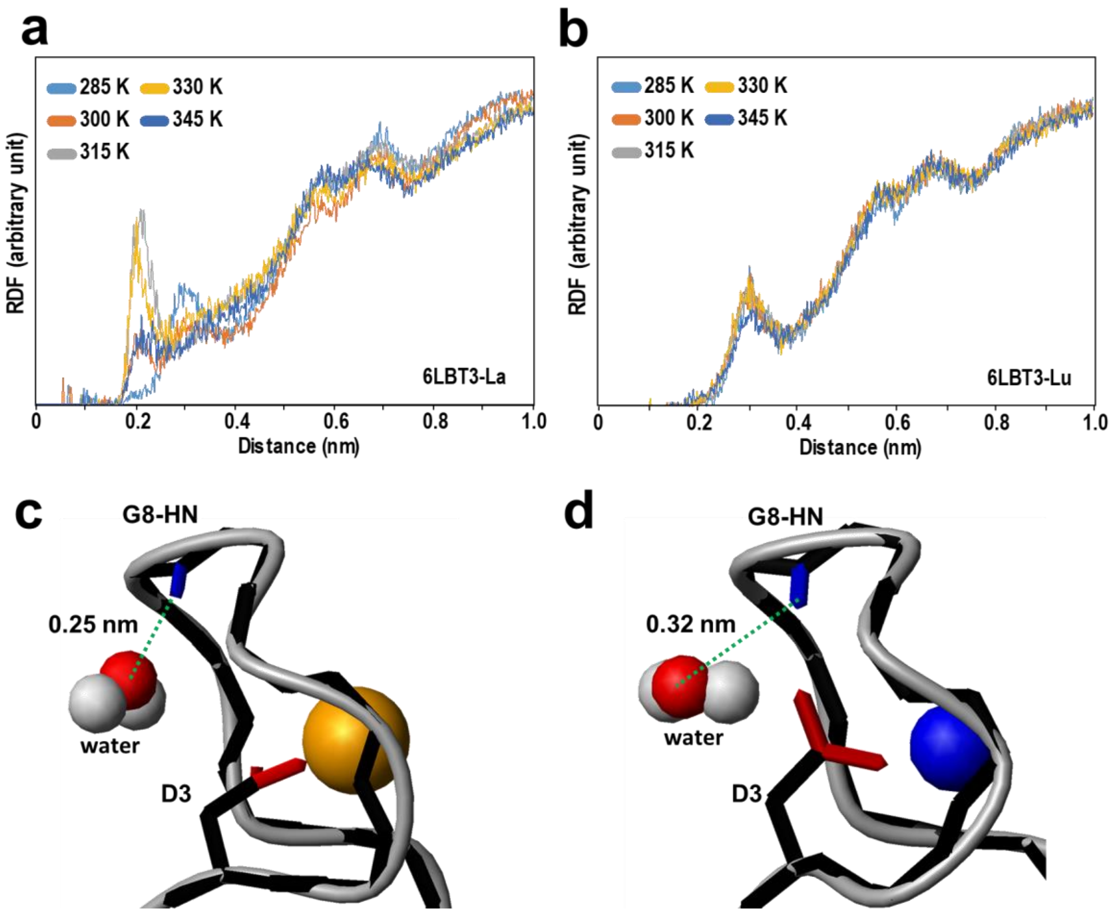

Figure S11. RDF of water molecules around G8-HN.

(a, b) Comparison of RDF of water molecules around G8-HN between (a) 6LBT3-La and (b) 6LBT3-Lu. The horizontal axis indicates the distance between G8-HN and the oxygen atom of a water molecule. (c, d) A structural example of (c) bidentate chelation or (d) monodentate chelation of D3 carboxylate. The hydrogen atoms of the peptide (except for G8-HN), amino acid sidechains (except for D3), and backbone carbonyl oxygens were omitted for clarity. The orange and blue spheres indicate La^{3+} and Lu^{3+}, respectively.

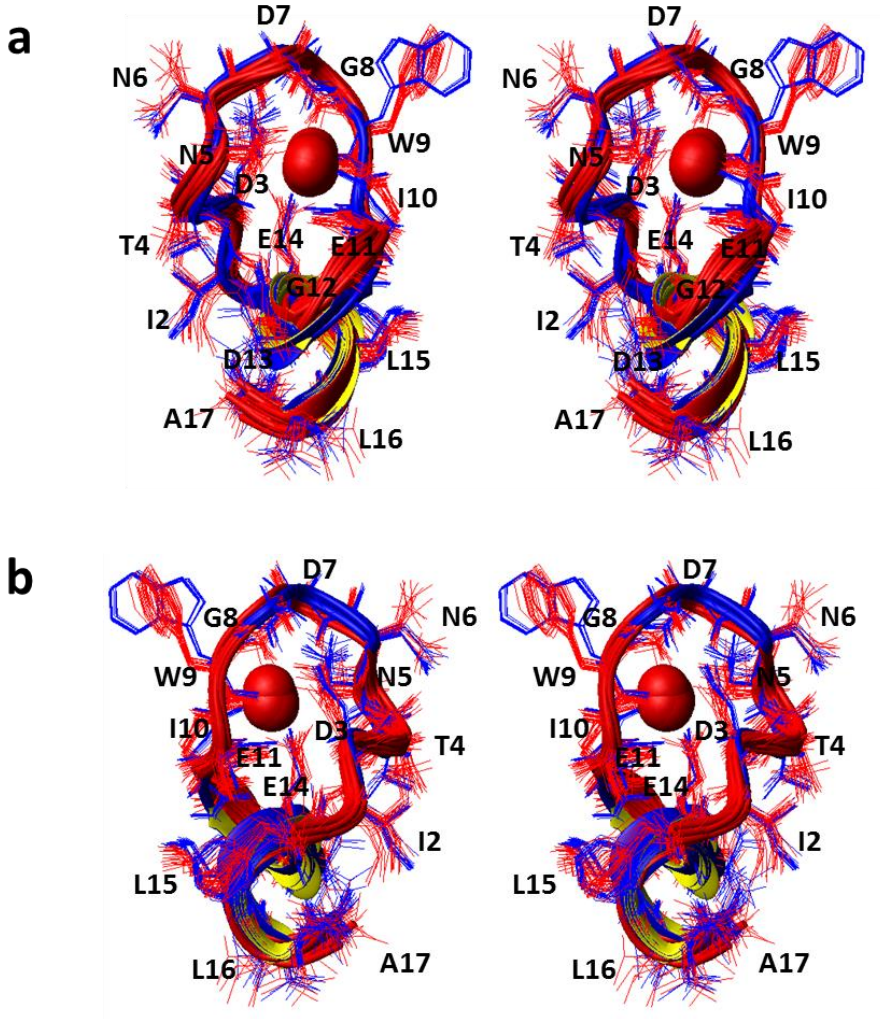

Figure S12. Stereoviews of the structures of the complexes 5LBT3-La and 5LBT3-Lu. a) 5LBT3-La (red) and 5LBT3-Lu (blue) are superimposed. The sidechain protons have been removed to simplify the images. b) 180° rotated view of a).

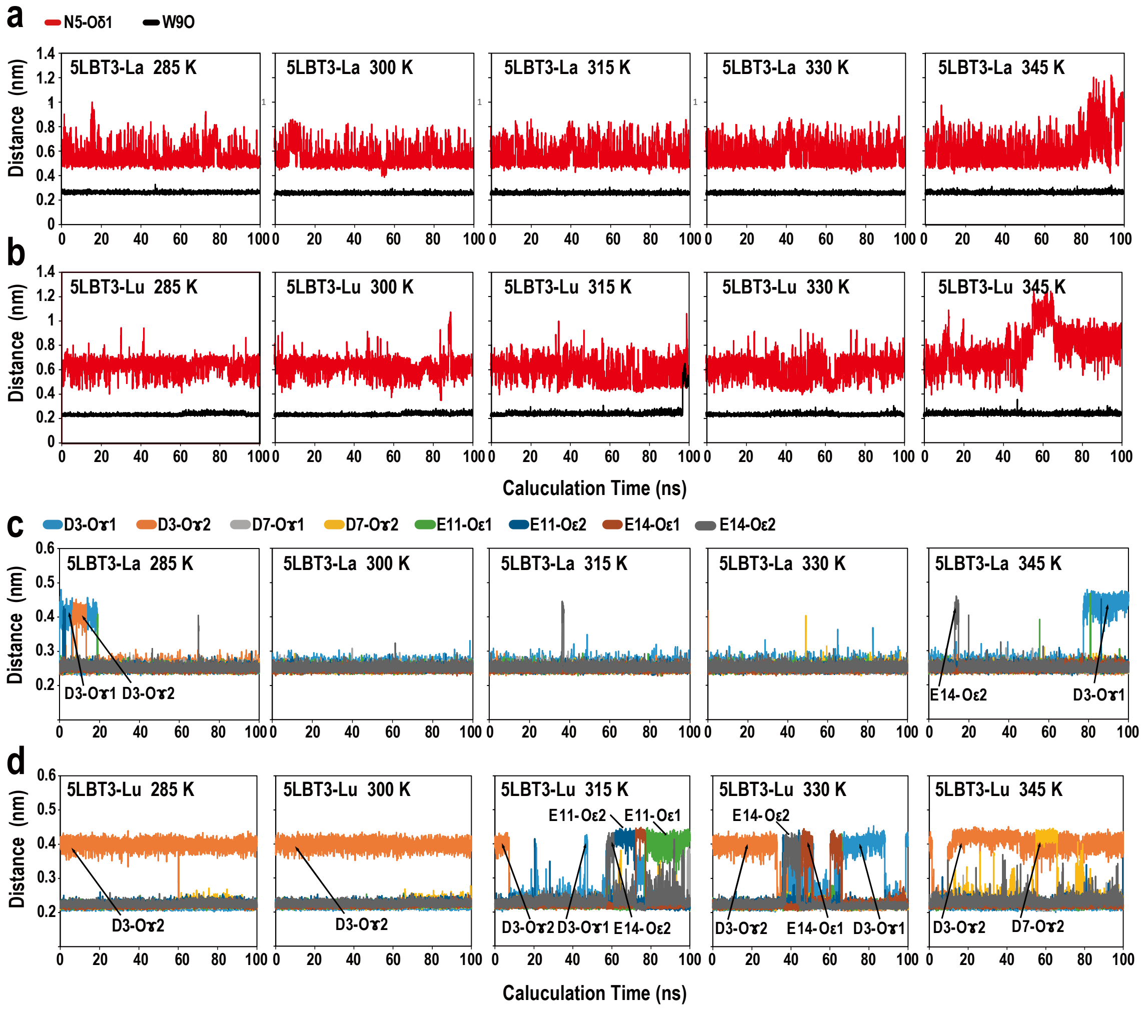

Figure S13. Changes in the distances between Ln^{3+} and coordinated oxygens during MD simulations of the 5LBT3-Ln system. (a,b) Changes in the distances between Ln^{3+} and the coordinated carbonyl oxygens N5-Oδ1 and W9O. (c, d) Changes in the distances between Ln^{3+} and the coordinated carboxylate oxygens D3-Oγ1, D3-Oγ2, D7-Oγ1, D7-Oγ2, E11-Oε1, E11-Oε2, E14-Oε1, and E14-Oε2. (a) and (c) represent the 5LBT3-La system, and (b) and (d) represent the 5LBT3-Lu system. The simulations were conducted at different temperatures (285 K to 345 K), as indicated in each panel. The dissociation of the carboxylate groups was not observed under any conditions. The carboxylate groups maintained bidentate chelation or monodentate chelation with the Ln^{3+} throughout the calculation.

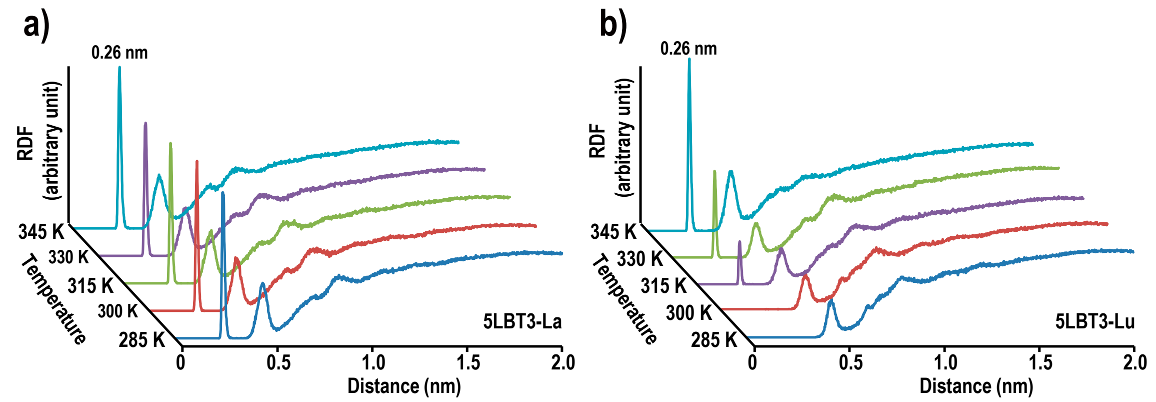

Figure S14. RDF of water molecule around 5LBT3-Ln.

RDF of water molecule around 5LBT3-La (a) and 5LBT3-Lu (b). Horizontal axis indicates the distance between Ln^{3+} and an oxygen atom of a water molecule. The peak at 0.26 nm indicates the existence of a water molecule that directly coordinates Ln^{3+}.

Table S1. Thermodynamic parameters for the reaction of LBT3 with Ln^{3+}.

	La	Nd	Sm	Gd	Tb
**N**	**1.01 ± 0.05**	**1.36 ± 0.09**	**1.05 ± 0.04**	**1.01 ± 0.08**	**0.96 ± 0.06**
**Δ*G* [kcal/mol]**	**-6.82 ± 0.03**	**-8.33 ± 0.07**	**-8.65 ± 0.17**	**-8.96 ± 0.10**	**-9.19 ± 0.09**
**Δ*H* [kcal/mol]**	**3.83 ± 0.28**	**2.00 ± 0.04**	**1.63 ± 0.12**	**1.97 ± 0.17**	**2.06 ± 0.08**
**Δ*S* [cal/mol/deg]**	**37.60 ± 0.91**	**36.50 ± 0.23**	**36.30 ± 0.40**	**38.63 ± 0.47**	**39.73 ± 0.18**
**-*T*Δ*S* [kcal/mol]**	**-10.65 ± 0.26**	**-10.33 ± 0.07**	**-10.28 ± 0.11**	**-10.94 ± 0.13**	**-11.25 ± 0.05**

	Dy	Ho	Er	Yb	Lu
**N**	**0.97 ± 0.08**	**0.91 ± 0.03**	**1.01 ± 0.06**	**0.97 ± 0.02**	**1.04 ± 0.07**
**Δ*G* [kcal/mol]**	**-9.18 ± 0.11**	**-9.23 ± 0.06**	**-9.16 ± 0.05**	**-9.20 ± 0.05**	**-8.97 ± 0.12**
**Δ*H* [kcal/mol]**	**2.24 ± 0.12**	**2.43 ± 0.10**	**2.51 ± 0.18**	**2.91 ± 0.04**	**2.51 ± 0.08**
**Δ*S* [cal/mol/deg]**	**40.34 ± 0.61**	**41.18 ± 0.42**	**41.23 ± 0.66**	**42.77 ± 0.29**	**40.54 ± 0.31**
**-*T*Δ*S* [kcal/mol]**	**-11.42 ± 0.17**	**-11.66 ± 0.12**	**-11.67 ± 0.19**	**-12.11 ± 0.08**	**-11.48 ± 0.09**

N: reaction stoichiometry.

T: temperature.

The experiment was performed at 10 °C.

The data indicate the mean value of experiments performed at least in triplicate.
